# Atrophy of the Teeth

**Published:** 1875-02

**Authors:** N. Marshall Burkholder

**Affiliations:** Harrisonburg, Va.


### ARTICLE II.

### *Atrophy of the Teeth.*

BY N. MARSHALL BURKHOLDER, D. D. S., HARRISONBURG, VA.

Read before the Virginia State Dental Association.

In 1872, Mr. C. brought his daughter, aged 21 years, to me to see what could be done for her mouth.

Upon examination, I found the teeth, 29 of which were present, to present the worst case of what was apparently Atrophy I had ever seen, and also the most unsightly appearance. I extracted them all, and, by the way, at one sitting, without anæsthetics, simply giving one wine glass of whiskey, and have them now before me mounted in plaster and articulated.

Upon opening the mouth, perhaps the first thing which would be noticed is the uniform dirty brown color of the teeth. The crowns are of medium size and well shaped. The fangs are long, very white and well developed; and the articulation and general arrangement good.

The peculiar feature of the case is that with the exception of several carious points or proximal surfaces, and these not of a serious character, the teeth are nowhere affected with disease or loss of structure save on their cutting edges and grinding surfaces and extending over their sides, uniformly, about one line or more around each tooth. Here a denuding process has been at work diverting the crowns of enamel in its progress, the enamel terminating at its margin with a more or less abrupt shoulder around the body of the teeth, showing its varying thickness; and the dentine, thus pealed as it were of its coat, standing alone.

On some of the teeth shrivelled enamel is left in part on the central portions of the grinding surfaces, and a close examination would seem to show the destructive process has been slower here, its ravages being more extensive, and uniformly so, on the cusps and just outside the margin of the grinding surfaces a uniform distance. On several of the oral teeth it has affected chiefly the anterior surfaces; but in most cases it has extended entirely around the teeth.

Upon inquiry, the parent, who in appearance is a stoutly built, sinewy, laboring man, stated that the teeth possessed their present dirty brown color when erupted. Other members of his family are similarly affected. His grandmother had such teeth. There are traces of it in his own mouth. There is abundant evidence of the scrofulous and rheumatic diatheses in the young lady's ancestors.

We have here plainly the result of an imperfect enamel formative process, aggravated by denudation since eruption, in which, doubtless acidulated oral secretions played a prominent part.

And whatever may be the proximate cause of the defective enamel formation, it would appear beyond all doubt to be connected with and the remote effect of hereditary trouble. But certain features of the case under so general a statement extremely unsatisfactory. Indeed it only serves as a sort of stand point, from which one casts his eyes about with strong desire to know something of the ætiology of the case.

In the ordinary cases of Atrophy, let us say, for instance, of the variety of shrivelled pits in two or more teeth, with which every practitioner often meets, we are accustomed to be comparatively well satisfied if we can point out the fact that at a certain period in infancy, a constitutional disturbance interrupted the nutrition of the organ, either by a minus supply of lime salts, or an inability to appropriate the needed material. And that the shrivelled appearance of the structure is the direct natural result of this cessation, the normal formation being resumed on the subsidence of the constitutional affection. Especially so when our judgment of the time of this trouble is verified by the parent. It is quite possible we are too easily satisfied with the general definitions which Dental Science can only at present offer. Nevertheless, in that ease there seems to be in the cause assigned a complete adaptation to the production of the anatomical results which we find. True, we are still comparatively ignorant as to what are the precise changes of the structural constituents of the calcifying tooth which underlie this pathological condition. A generally received axiom now, however, as the result of the medical investigations of our times, to the identity of pathological phenomena with the phenomena of physiological life. "An examination of the anonalises of the osseous system, says Rindfleisch, reveals the fact that a large number of the diseases of the bone depend upon a simple plus or minus of normal growth. A far greater part depends upon the excessive prominence of individual anatomical forces which play a subordinate role in normal growth." So that a knowledge of the normal formation of bone from periosteum, and of the transformation of cartilage into bone, gives one the foundation of the pathological histology, or *minute abnormal structural changes* of the osseous system.

With this key it is to be hoped that the study of the histogenesis of the teeth, both normal and pathological, may be prosecuted to a point as satisfactory and as thorough as in the nature of things it may be. But while in this case we have these common difficulties, there is apparently another and a coarser question added, pointing it would seem to a prominent local link. And it is this to which I here more particularly refer.

The question arises how we are to account for the production of enamel of inferior quality *uniformly* on the faces, cutting edges or prominent portions of the teeth, where at the age of 21 it is almost wholly destroyed, while the remaining portions clearly prove their better quality, in that they have successfully resisted all destructive agencies.

It is possible that an abnormal condition of the preformative membrane of Raschkow, which doubtless constitutes the bond of union between the enamel fibres and the dentine, may end subsequently in its loss, and thus favor the denuding process referred to, and account for the enamel color. But how vile this account for other and more prominent features of the case. Without however, entering into a review of the several causes of such a condition as may be suggested by those authors with which I am familiar, let it suffice here to say, that none of them appear to be adapted to the production of combined and uniform results, such as are exhibited in this case.

Is it possible that a premature attempt at root formation, analogues to premature ossification in bone which these produces disturbances in normal development, or rather, perhaps, a tardy enamel formation, or both may be a local or external cause of this diseased condition ?

To be brief, that the formation of cementum by that elongated portion of the preformative membrane apparently invested with this office, taking place simultaneously with a late commencement of the enamel process, owing to constitutional dyscrasia, and consequently the pressure of the teeth, however slightly, upon their walls during the incipient stages of enamel formation, may as a local cause so affect the young tissue on its face or front to produce these effects.

It would seem to be possible to have a disturbance in normal development here attended with an upward movement of the whole delicate structure, by the construction of the base of the dental sac, it may be; and that such disturbances may, in varying degree of intensity and continuance be the parent of various forms of disease in these organs.

It is deserving of some consideration in this connection that the effect of pressure on the occipital bone in rachitis is to impede and even in places to prevent ossification.

My mind has been led to the theory adduced by such considerations as these, viz:—

1. There are no continued or repeated constitutional disease, such as measles, scarlatina, etc., to account for such results.

2. If even it were conceded that repeated constitutional troubles were present sufficient to account for it, it would prove too much. It would leave unexplained the fact that the cutting edges, apices or most prominent positions of enamel, (commonly the least liable to loss because prominent,) are most defective, and lost, while those portions most liable to destructive processes remain sound.

2. Notwithstanding the different periods of development running through a term of years, we have the cutting faces or apices alone affected, and uniformly so.

4. It would seem that there must have been a common cause to act upon all alike, after some invariable law, one uniformly acting at the commencement of the enamel formation of each tooth, and acting upon a certain circular, well defined and limited portion of the enamel membrane, and to a point on the side of the tooth equi-distant from the apex in all.

5. The defective portion embraces only the whole actual face or front of the tooth in advancing, *i. e.* the grinding surface and side swell.

6. The confinement of defect almost wholly to the anterior surfaces of some may result from the inclination and advancement of such teeth outwards as well as upwards. The molars and bicuspids are for the most part wholly encircled, and also the cuspidati.

---

### SELECTED ARTICLES.

